# Interaction of Different Drying Methods and Storage on Appearance, Surface Structure, Energy, and Quality of *Berberis vulgaris* var. asperma

**DOI:** 10.3390/foods11193003

**Published:** 2022-09-27

**Authors:** Seyede Laleh Talebzadeh, Hamideh Fatemi, Majid Azizi, Mohammad Kaveh, Amirali Salavati Nik, Mariusz Szymanek, Ryszard Kulig

**Affiliations:** 1 Department of Horticultural Science, Agricultural Faculty, Ferdowsi University of Mashhad, Mashhad 9177948974, Iran; 2 Department of Petroleum Engineering, College of Engineering, Knowledge University, Erbil 44001, Iraq; 3 Department of Agricultural, Forest and Transport Machinery, University of Life Sciences in Lublin, Głęboka 28, 20-612 Lublin, Poland; 4 Department of Food Engineering and Machines, University of Life Sciences in Lublin, Głęboka 28, 20-612 Lublin, Poland

**Keywords:** drying method, energy, phenolic compound, storage, surface structure

## Abstract

Seedless barberry fruit is native small fruit in Iran. To examine the impact of various drying methods and storage on the biochemical attributes (Vitamin C, Anthocyanin, Phenol, pH, TA), color index (a*, b*, L*, ab, and Chroma), drying time, and fruit microstructure (by SEM) of seedless barberry (*Berberis vulgaris* var. asperma), and effective moisture diffusivity coefficient (D_eff_), specific energy consumption (SEC), energy efficiency (EE) of the dryers, this experiment was performed. Drying treatments include microwave (100, 170, and 270 W), oven (60 and 70 °C), cabinet (50 and 70 °C), shade, sun, and fresh samples (control) and storage 6 months after drying (in polyethylene packaging and at a temperature of 5–10 °C). Results showed minimum and maximum drying times (50 min and 696 h), were related to microwave (270 W) and shade methods, respectively. The highest color values were observed in fruits treated with control, shade and sun treatments and the lowest values were observed in cabinet (70 °C) methods. According to the SEM results, microwave significantly affected surface structure of the dried sample compared to others. The findings indicated that the use of artificial drying methods than natural methods (sun and shade) cause a more significant reduction in color indexes, while vitamin C, soluble solids, and anthocyanin were significantly maintained at a high level. Storage reduced anthocyanin content of fruits almost 12%. Moreover, it was discovered EE and SEC values varied in the range of 1.16–25.26% and 12.20–1182 MJ/kg, respectively. D_eff_ values were higher in microwave 270 W.

## 1. Introduction

The *Berberis* sp. is native to Asia, Europe, Africa, North America, and South America, including east areas of Iran with over 11,000 hectares of land under cultivation. Iran is the world’s largest producer of *B. vulgaris* fruits [1]. This genus is extensively used in traditional medicine to treat stomach and duodenal ulcers, persistent diarrhea, and rheumatic joint diseases [2]. Also, Fresh fruit is used to make jellies, syrups, jams, sauces, juices, fruit concentrates, and carbonated beverages and dried fruit is primarily utilized as a food component in Iranian recipes [3].

Traditional and industrial methods are two broad categories of drying methods [4]. Traditional methods are time-consuming; for example, in drying with the sun outer layer of the products are initially dry and forms a layer of poor heat conduction, which prevents drying speed. In addition, there is a high risk of increased microbial load in food. Therefore, these methods have severe concerns about long drying time, generally low quality, and high energy consumption [5]. In contrast, industrial drying not only keeps the food at an acceptable quality but also decreased the drying time [6,7]. Various drying processes for dehydrating vegetal products have been developed and employed. Cabinet drying, spray drying, freeze drying, microwave drying, and osmotic dehydration are the most common procedures used to dry vegetal products [8].

Microwaves, as a kind of electromagnetic wave, can be used in the dehydration of fruit and vegetables. Under the influence of microwaves, the polar structure of water molecules in fruits and vegetables vibrates at a high frequency, resulting in a significant effect and thermal motion, allowing moisture to be lost quickly [9,10]. Compared to traditional cabinet, microwave drying offers many advantages for use in the food drying process, including volumetric heating, high thermal efficiency, quicker drying times, and increased product quality. In some studies, they have combined some drying methods together to take advantage of their advantages for drying [11].

Numerous studies confirmed that agricultural practices affect qualitative, thermal nutritional properties of various agricultural products during production and drying using different desiccants, including onion [12,13], potato [14], ginger [15], apple slices [16], spearmint [17], green beans [18], lemon verbena [19,20] and broccoli [4] were performed. Regardless of the drying methods, fruits’ high moisture content significantly negatively impacts their storage. Decreased water levels can impact the ultimate crop’s flavor, color, nutritional value, size, weight, and shape [21,22]. The samples’ characteristics change during drying due to heat and mass transfer from the product, as well as chemical interactions [23]. However, to increase shelf life, permit transportation, retain quality, and lower post-harvest loss, moisture reduction or drying through the simultaneous transfer of mass and heat is frequently used in the manufacturing of dried fruits and vegetables [24]. Also, the ultimate quality of the dried product, which is the overall qualities and properties of food that can please the consumer, is one of the most significant indications to consider during the drying process. There are also physical characteristics like shape, color, and texture, as well as nutritional properties like vitamins, pigments, and chemicals that give foods their antioxidant effects.

Given the medicinal value of seedless barberry fruit and the necessity for correct processing, proper drying methods can enhance the quality of the finished product while consuming less time and energy during the drying process. This study aims to examine and discuss the effects of different drying methods on drying time, biochemical factors, and quality of seedless barberry during drying and storage.

## 2. Materials and Methods

### 2.1. Treatments

The fruit clusters and shrubs of the seedless barberry variety asperma were harvested. Shape, size, and colour all had an impact in selection In Ghaenat city, South-Khorasan province, Iran. Hand-harvested mature fruits from four randomly selected branches of each 17-year-old bush. To determine the initial moisture content, 3 samples of seedless barberry (100 g) were put in oven (105 °C, 24 h). Moisture content was calculated based on plant dry weight. Moisture content is determined from the below equation [25].

MC_d_._b_. = W_i_ − W_d_/W_d_ (1)

where, W_i_ is the initial mass of product (kg) and W_d_ is the Mass of dried product (kg).

Drying treatments include sun, shade 25 °C, cabinet 50 (H 50) and 70 °C (H70), oven 60 (O 60) and 70 °C (O70), microwave 100 (M 100), 170 (M170), and 270 W (M270), and fresh fruit as control (Table 1). A hundred-gram fruits divided for each replication and weight during a specific time until 27% of fresh weight.

After the drying process and the completion of the first stage of measuring the appearance and biochemical factors of the dried treatments, each of the samples were kept in polyethylene packaging in the storage environment; 5–10 °C for 6 months.

### 2.2. Drying Time

The initial moisture content of seedless barberry fruit was 65% (w.b.), which should be reduced to 27% (w.b.). The drying time of the samples in different drying treatments until their moisture content reached 27% was calculated [26].

### 2.3. Color

The color indices of the samples were measured based on the components a*, b* and L* and ab by the colorimeter HUNTER LAB (model 0.45) [27].

L* indicators indicate dark or light color, ranging from L* = 0 (dark) to L* = 100 (white). Indicators a*+ [28] and −a* (green), +b* (yellow), −b* (blue). Chroma or C describes luminosity, intensity, degree of color purity.

C = [(a*)^2^ + (b*)^2^]^1/2^ (2)

### 2.4. SEM Imaging

Images from fruit surfaces were captured (magnification of 500× and 1000×) using a scanning electron microscopy model LEO 1450VP (Variable Pressure), made in Germany, with a maximum voltage (KV) of 35, was used. Sputter coater (Au-Pd), model SC7620, made in England, was used to prepare the samples. Seedless barberry samples were coated with gold and palladium for 2 min

### 2.5. Anthocyanin Content

Wagner [29] method was used to measure the content of anthocyanins. Fresh seedless barberry fruit (0.1 g, 3 replication) and 10 mL of methanol acid were completely pulverized in a mortar (methanol: hydrochloric acid; ratio of 1:99) and the extract was poured into a screw test tube and kept in the refrigerator for 24 h. Then supernatant’s absorbance was calculated at 512 nm by a spectrophotometer (UV/Vis, Shimadzo, model 2502). The following formula was used to determine the concentration: A = εbc. In this equation, A represents the adsorption, b is the cell’s width (cm), ε is Molar absorption coefficient and c is the desired solution’s concentration. The final concentration of anthocyanin represented as µg/gDW.

### 2.6. Total Phenol Content (TPC)

TPC was measured according to Samadi [30] methods. The methanolic extract was prepared with 100 mg of plant samples (3 replication). Then 300 µL of the extract was added to 1.2 mL of 7.5% sodium carbonate and 1.5 mL of 10% Folin-Ciocalteu. The reaction mixtures were placed in the dark for 30 min and then were read at 765 nm via spectrophotometer (UV/Vis, Shimadzo, model 2502) and compared to a gallic acid calibration curve to estimate the mg of Gallic acid/g extract. For blank, all process was the same and just we added 300 µL methanol instead of plant extract [30].

### 2.7. pH, TSS, and Titratable Acidity (TA)

For preparing before and after drying 5 g fruits (3 replication) grind via pestle and mortar and added to water and then put in shaker (15 min/50 rpm A digital refractometer (model PR101, Atago [0–45 percent] Co., Ltd., Tokyo, Japan) was used at room temperature and then the amount of 10 to 20 mL of fruit extract is measured by the pH meter. A refractometer was used to measure the total soluble solids (TSS). Fruit extract (5 mL) diluted with distillate water to 100 mL. TA as determined titration with NaOH 0.1 N at pH 8.23.

### 2.8. Vitamin C Content

First, 10 mL of fruit juice, 2 mL of starch as an indicator and 20 mL of distilled water were mixed. Titration with 0.01 N iodine dye solution continued until the color changed to blue. Then the volume of iodine consumption was recorded as 100 mg of vitamin C per 100 mL the sample was calculated according to the following equitation [31]:

Vitamin C (mg/100 cc) = volume of iodin ∗ 0.88/sample volume ∗ 100 (3)

### 2.9. Effective Moisture Diffusivity Coefficient

Assuming that moisture transport is only through diffusion along the radial direction, and the drying process was conducted at a relatively long time, the analytical solution of the Fick second low in unstable diffusion for spherical materials can describe the moisture displacement during the drying process as shown (4) [26]:

(4) $$MR=\frac{M_{t}-M_{e}}{M_{b}-M_{e}}=\frac{6}{\pi^{2}}\sum_{n=1}^{\infty }\frac{1}{2n^{2}}\exp\left(-n^{2}\pi^{2}\frac{D_{eff}t}{r_{e}{}^{2}}\right)$$

where *M_b_* is initial moisture content, *M_e_* is equilibrium moisture, and *M_t_* is the moisture of the berberis at any time during the drying. For products with high initial moisture content. For long-time drying, Equation (4) can be simplified to Equation (5):

(5) $$MR=\left(\frac{6}{\pi^{2}}\right)\exp\left(-\frac{\pi^{2}D_{eff}t}{r_{e}{}^{2}}\right)$$

where *r_e_* is the radius of berberis seed (m), *n* is the index ranging from 1 to infinity, *t* is the drying time (s), and D_eff_ is the effective diffusivity coefficient (m^2^/s).

*K*_1_ could be calculated by plotting ln(D_eff_) vs. time as shown in Equation (5). Thus, D_eff_ can be determined by:

(6) $$K_{1}=\left(\frac{D_{eff}\pi^{2}}{r_{e}{}^{2}}\right)$$

### 2.10. Specific Energy Consumption (SEC)

The SEC of each drying condition can be expressed considering the drying time and the energy applied by different components of the microwave, cabinet and oven. In other words, the SEC of these cabinets can be defined as the energy required for evaporating one unit mass of moisture which includes the thermal energy, blower, engine, and magnetron. It can be estimated by the following Equations [32]:

SEC= Total energy supplied in drying process/Amount of water removed during drying(7)

### 2.11. Energy Efficiency (EE)

The EE was determined by Equation (8) [33]:

ή_e_ = E_eva_/SEC, (8)

E_eva_ = h_f_._g_.m_w_, (9)

where ή_e_ is the EE (%), E_eva_ is the energy required to evaporate moisture (kJ) and h_f_._g_ is the latent heat of vaporization (kJ/kg).

In Equation (10), h_f_._g_ shows the latent evaporation heat (kJ/kg) which is calculated as a function of absolute temperature (T_abs_ K) [32]:

h_f_._g_ = (7.33 × 10^6^ − 16 T_abs_^2^)^0.5^, 273.16 < T_abs_ < 338.72 h_f_._g_ = (2.503 × 10^3^ − 2.386 (T_abs_ − 273.16), 337.72 < T_abs_ < 533.16 (10)

### 2.12. Statistical Analysis

The experimental design was factorial based on a completely randomized design with three replications (n = 3). All the measurements were made at least in triplicates. Statistical analysis and comparison of the mean of the obtained data were performed using JMP 8.0 statistical software. Means were compared using ANOVA, followed by LSD test (* *p* ≤ 0.05).

## 3. Results and Discussion

### 3.1. Drying Time

**Figure 1:**
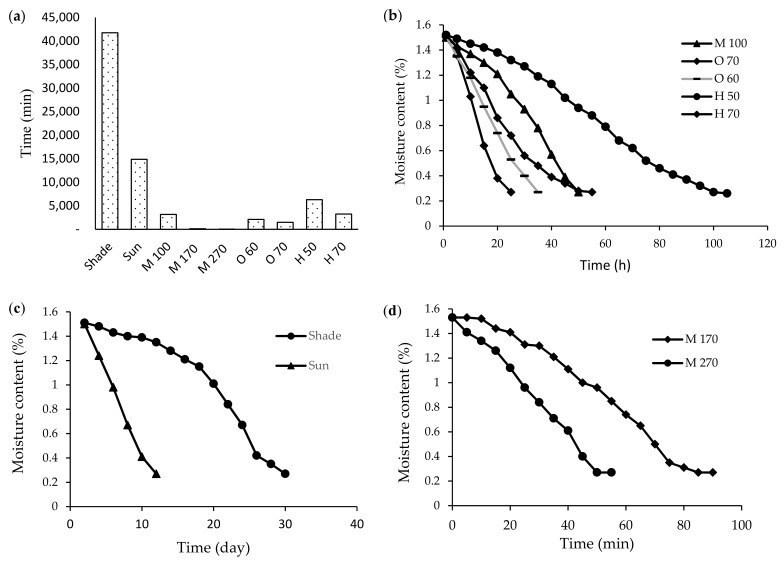
Effect of various drying techniques on the drying time of seedless barberry. (**a**) Drying time of different methods, (**b**) Moisture content curve of different methods (microwave, oven and cabinet), (**c**) Moisture content curve of shade and sun dryer, (**d**) Moisture content curve of microwave treatments (170 W and 270 W). Microwave 100 W (M 100), Microwave 170 W (M 170), Microwave 270 W (M 270), Oven 60 °C (O 60), Oven 70 °C (O 70), Cabinet 50 °C (H 50), Cabinet 70 °C (H 70).

Figure 1a show that various drying techniques result in noticeably varying drying times. The shade treatment required the most time (696 h), whereas the microwave 270 W treatment took the shortest time (50 min). Due to different temperatures, the oven drying times varied between 1537.3 to 2142.6 min. This time in cabinet methods went between 54 to 105 h. The results show that increasing oven temperature and cabinet rises the drying curve’s slope. In natural drying methods, sun-dried treatments required 248 h and shade-dried treatments took 696 h to achieve the desired moisture content (Figure 1c). Microwave drying times from 50 min and 120 min (respectively at 170 and 270 W), (Figure 1d) to 53 h (3180 min at 100 W), were variable.

Compared to other ways, microwave drying speeds up the process because the microwave polarizes the water molecules inside the samples, resulting in increased heat inside the product and increasing the internal vapor pressure in the sample [34]. When the sample’s cellular tissue eventually swells, it creates more holes, which makes it easier for moisture to escape and shortens the drying time. Additionally, cabinet drying dries the product’s outer layer first, which reduces the material’s surface area and permeability. By adding a hard layer to the food’s surface, moisture cannot continue to escape and it can prolong the drying time [35]. An et al. [36] investigated the drying of ginger by different methods (hot air and infrared, freeze dryer, microwave, and microwave- hot air combination). The results showed that the use of the combined microwave compared to others reduced the drying time; for example, applying a combined microwave- hot air 88% compared to hot air speeds up the exit of moisture from the product, and consequently the drying time was shorter.

### 3.2. Color

According our results showed significant differences (* *p* ≤ 0.05) in all traits such as a*, b*, L*, ab and chroma (Table 2).

**Figure 2:**
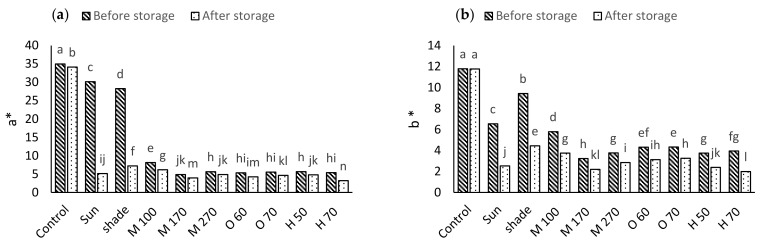

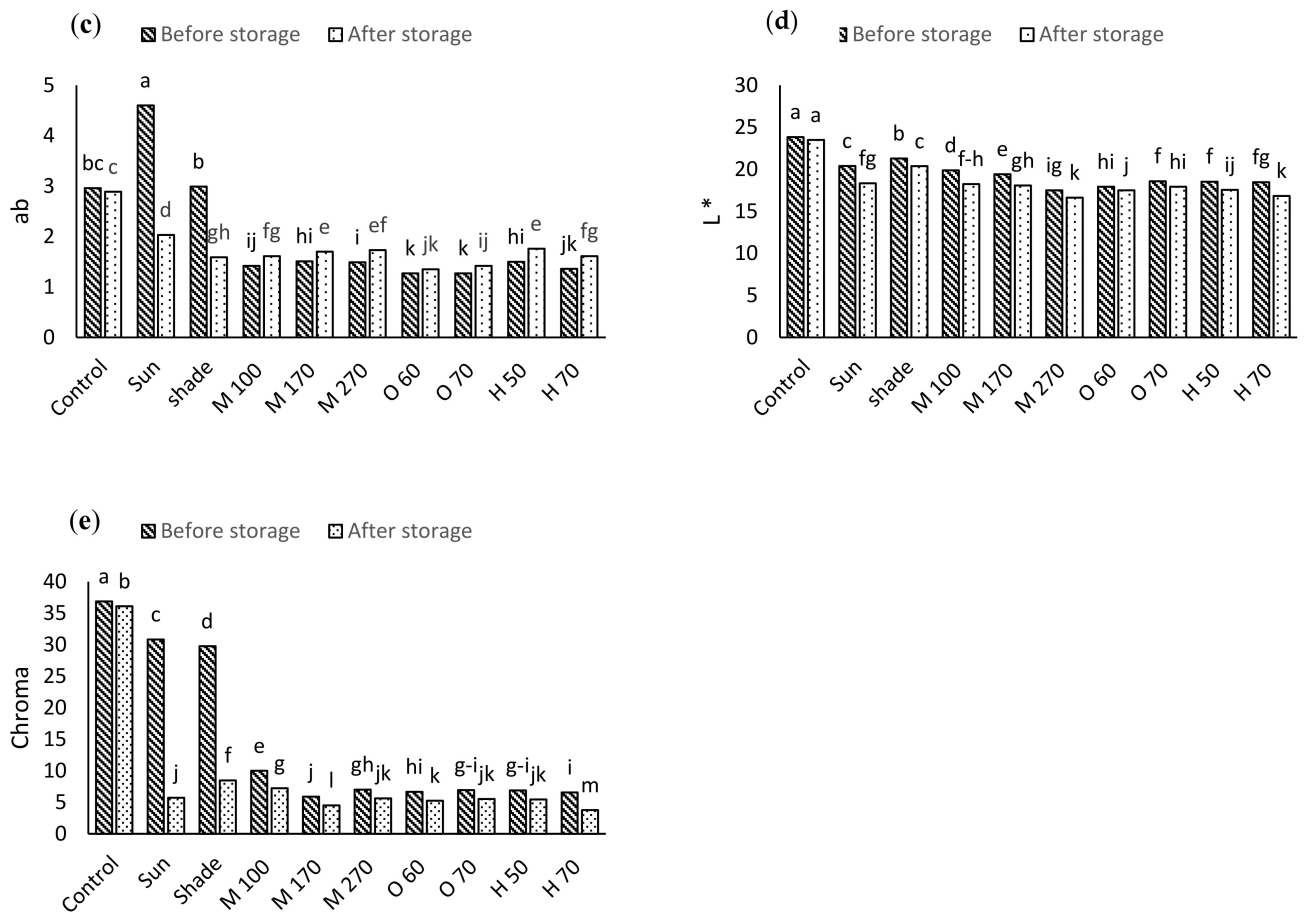
Effect of different drying methods on color indexes (**a**–**e**) (a*, b*, ab, L*, and chroma) of Seedless barberry fruits. Microwave 100 W (M 100), Microwave 170 W (M 170), Microwave 270 W (M 270), Oven 60 °C (O 60), Oven 70 °C (O 70), Cabinet 50 °C (H 50), Cabinet 70 °C (H 70). The LSD test shows that bars with various letters are substantially different from one another at *p* ≤ 0.05.

Among the parameters studied for the color index, the parameter a* in the storage period showed the most changes compared to other parameters (Figure 2a). The initial value of parameter a was equal to 34.56. This parameter decreased during drying in all treatments. The intensity of these changes was greater in microwave powers 270 W, oven and cabinet treatments. The initial value of b* was 11.77 (Figure 2b). The rate of reduction of b* parameter was higher in cabinet and microwave treatments. The lowest ab factor was recorded in fruits dried via oven (Figure 2c). In the study greater decrease in L* factor was observed in microwave 270 W and cabinet (70 °C) (Figure 2d). After storage, the greatest reduction in the a* factor was observed in shade and sun treatments and then in microwave 100 W and cabinet (70 °C). For L* factor, there is a greater reduction in the amount of brightness under microwave 270 °C and cabinet 70 °C. Therefore, according to the parameter ab*, it is inferred that sun and shade treatments maintain the color index (red-to-yellow ratio) better than other treatments for drying seedless barberry fruit, which also preserves the appearance quality during storage. The highest amount of chroma index in both times after drying and after the storage period was observed in fresh samples and then in shade and sun treatments and the lowest amount was in the cabinet treatment at 70 °C. It seems that although the sun-dried and shade-dried treatments after drying had a more severe reduction in color factors until the end of the storage period, but due to the initial preservation of color characteristics during drying, compared to other drying treatments were more successful in preserving the color properties, due to the presence of heat in artificial drying methods, which is strongly involved in the destruction of seedless barberry pigments.

Kayacan et al. [37] reported in persimmons under hot air, more drying time can result in accelerated pigment deterioration and non-enzymatic browning. Additionally, drying time is shortened in the microwave treatments, and this is followed by the degradation of pigments from interaction of hot air and power. The microwave reduced amount of discoloration of the coriander fruit samples during the drying process [38]. In another study, Łechtańska et al. [39] on the drying of green pepper using different desiccants (hot air, microwave- hot air, microwave- hot air -infrared). The least color change was related to the microwave- hot air, due to less drying time.

### 3.3. Biochemical Traits

According to Table 3, different drying methods significantly affect pH, TA, TSS, Vitamin C, anthocyanin, and phenol content. Also, all treatment affects significantly via storage except pH, but interaction effect shows a significant effect just for pH, TA, Vitamin C, and anthocyanin.

**Figure 3:**
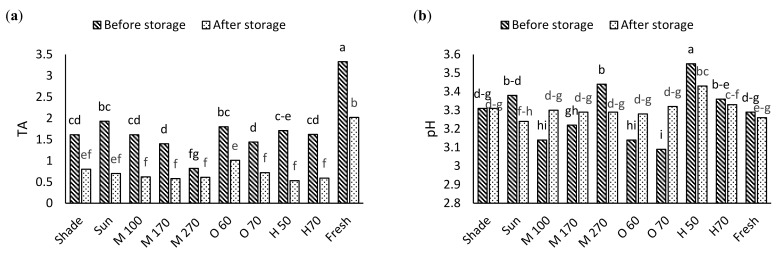

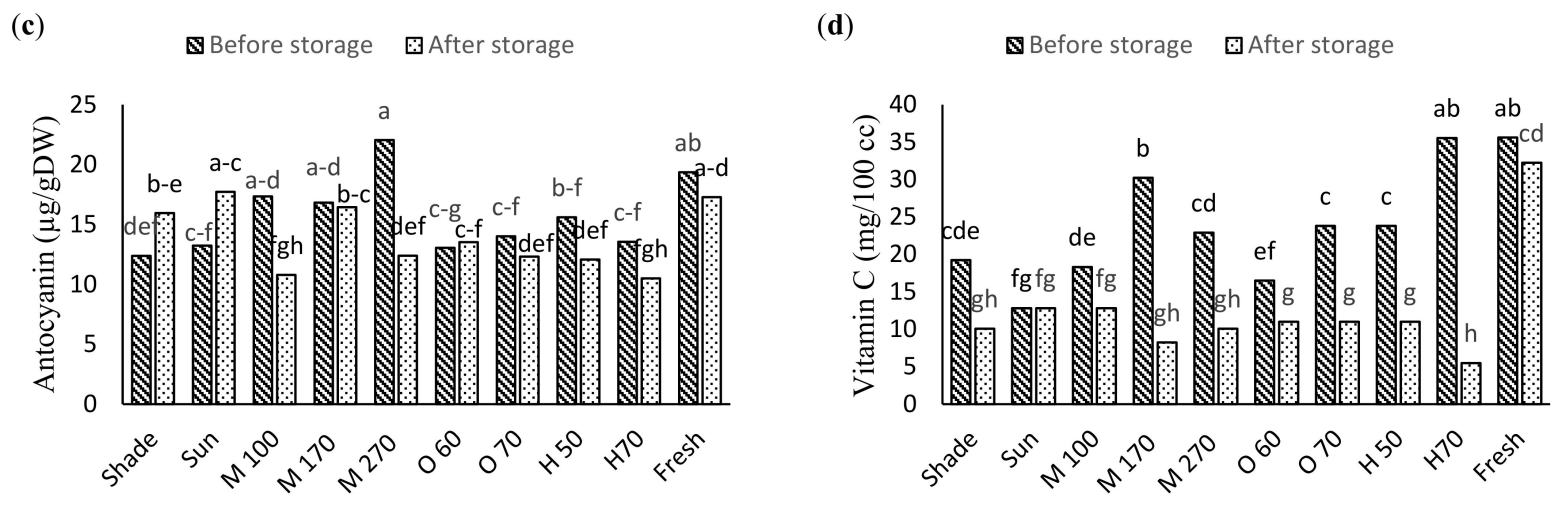
Effect of different drying methods on TA (**a**), pH (**b**), anthocyanin (**c**) and vitamin C (**d**), of Seedless barberry. Microwave 100 W (M 100), Microwave 170 W (M 170), Microwave 270 W (M 270), Oven 60 °C (O 60), Oven 70 °C (O 70), Cabinet 50 °C (H 50), Cabinet 70 °C (H 70). The LSD test shows that bars with various letters are substantially different from one another at *p* ≤ 0.05.

Also, comparing the average interaction effects of drying and storage on TA indicates that all drying treatments, before and after storage, have reduced the titratable acidity compared to the fresh sample (Figure 3a). Our results showed before storage with increasing temperature with oven and cabinet and also with increasing microwave power TA amounts decrease significantly. Figure 3b shows that pH in the 50 °C cabinet treatment was maximum amount (3.55), the lowest pH related to oven treatments (3.09 and 3.14) before storage. The browning reaction forms soluble and insoluble polymers when reducing sugars combine with protein amino acids or other nitrogenous substances. The decreasing of pH is a result of the loss of amino groups and the production of organic acids in this reaction [40].

The result shown cabinet and microwave have the highest level of TSS. Under storage conditions TSS was reduced. It seems that increasing the drying temperature in the case of oven and cabinet treatments and increasing the microwave power increases the TSS. The results of comparing vitamin C showed that the amount of vitamin C in seedless barberry fruit was affected by drying methods so that the amount of vitamin C in fresh fruit after harvest decreases over time and storage. According to Figure 3d, control obtained the highest vitamin C contents and then in the cabinet 70 °C and microwave at 170 W. In all the treatments, except sun-dried treatments, the amount of vitamin C was significantly reduced after the storage stage. However, sun-dried treatments, reduced ascorbic acid content compared to control treatments. Cabinet 70 ° C showed a significant reduction in the amount of vitamin C in the storage stage, despite the preservation of vitamin C during the drying time. Another scientist also reported storge has a significant effect on biochemical parameters of plants [20].

Anthocyanins content changed in different drying methods and also after storage period. Under storage anthocyanin level of seedless barberry fruit diminished in all dried treatments. In general, the highest amount of anthocyanin was observed in 270 W microwave treatment and fresh samples before storage (Figure 3c). After storage anthocyanin content of barberry fruit decreased in all dried treatments. It should be noted that the use of natural drying methods has increased the amount of anthocyanins.

Also, the comparison of the average interaction effects of drying and storage on phenolic compounds indicates that among the different drying treatments, only the 50 °C cabinet treatment shows a significant reduction in phenolic compounds compared to fresh seedless barberry samples. Phenolic compounds are reduced in different dried treatments under storage conditions. According to the result, it seems that the microwave treatments of 170 and 270 W and oven show the least amount of reduction in phenolic compounds. Microwave 100 W and cabinet 70 °C treatments had the highest reduction of phenolic compounds with 53.15% and 43.58%, respectively. Phenolic compounds, as active plant substances, have valuable antiviral, antimicrobial, antiviral, and anticancer properties [41]. Other scientists reported the same results in other plants such as drying has different effects on phenolic compounds and the antioxidant activity of different plants [42]. Microwave radiation generates very rapid and strong heat, according to Lim and Murtijaya, [43] which can cause catastrophic damage to polyphenolics. Furthermore, during the drying process, polyphenol oxidase and peroxidase activities can induce TPC loss, whereas Xu et al. [4] found that broccoli polyphenolics enhanced after the microwave procedure. They noted that during the microwave–hot-air drying process, unique energy is released that causes cellular elements to decompose and more polyphenolics to be secreted from the product texture. TPC, on the other hand, changes unevenly in different plant species and drying processes [44].

### 3.4. Surface Structure by SEM Image

**Figure 4:**
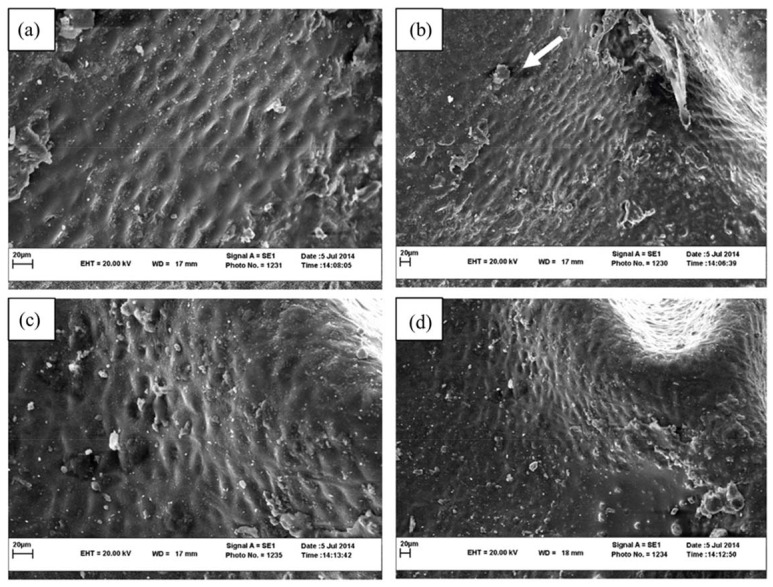
SEM images of Seedless barberry fruit surface structure under different drying methods, (**a**) Sun with magnification 1000; (**b**) Sun with magnification 500, (**c**) Shade with magnification 1000; (**d**) Shade with magnification 500.

Information about changes in plant microstructures enables us to control the drying process better and improve the product’s appearance. By observing the surface structure of the product, the effects of different drying methods and drying temperatures on microstructural changes of samples can be investigated. As we can see in Figure 4 drying seedless barberry fruit under shade, the surface of seedless barberry samples is less wrinkled, and the smooth texture of the fresh fruit is better preserved. Using the sun method causes the destruction of fruit tissue in some parts, as shown via the white arrow in the Figure 4b.

**Figure 5:**
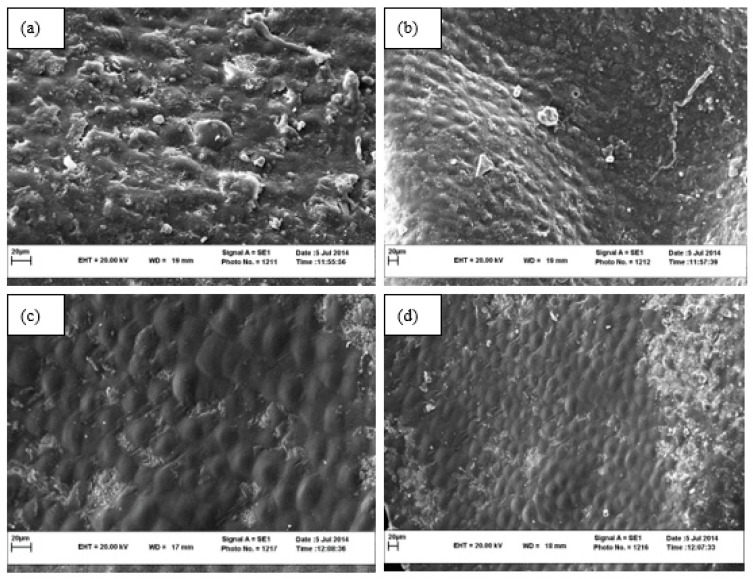
SEM images of seedless barberry fruit surface structure under different drying methods, (**a**) Cabinet 70 °C with magnification 1000; (**b**) Cabinet 70 °C with magnification 500, (**c**) Cabinet 50 °C with magnification 1000; (**d**) Cabinet 50 °C with magnification 500.

According to the result temperature affected surface structure significantly and drying under 70 °C (Figure 5a) increases the shrinkage of the fruit epidermis and destroys the fruit tissue compare to 50 °C (Figure 5c). These changes are quite evident in Figure 5 especially at 1000 magnification.

**Figure 6:**
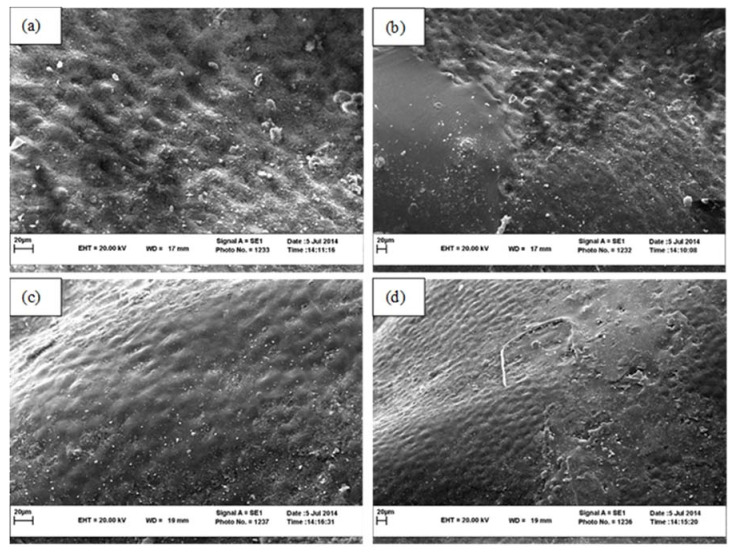
SEM images of Seedless barberry fruit surface structure under different drying methods, (**a**) Microwave 100 W with magnification 1000; (**b**) Microwave 100 W with magnification 500, (**c**) Microwave 270 W with magnification 1000; (**d**) Microwave 270 W with magnification 500.

**Figure 7:**
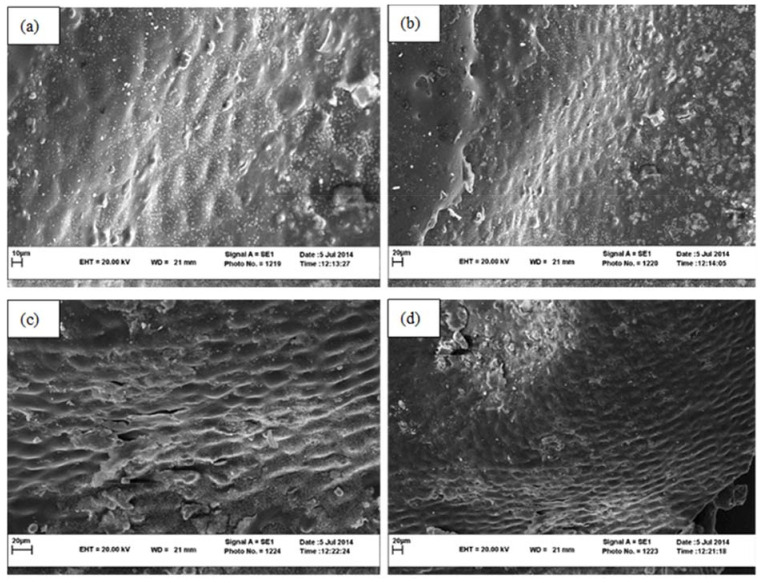
SEM images of Seedless barberry fruit surface structure under different drying methods, (**a**) Oven 60 °C with magnification 1000; (**b**) Oven 60 °C with magnification 500, (**c**) Oven 70 °C with magnification 1000; (**d**) Oven 70 °C with magnification 500.

According to the SEM images taken from the dried seedless barberry samples by different microwave powers (Figure 6), it is inferred that increasing the microwave power reduces the shrinkage of the seedless barberry fruit surface (Figure 6c). Due to the reduction of drying time at high power. According to the SEM imaging, (Figure 7) the highest reduction of seedless barberry fruits’ epiderm diameter was observed under Oven drying, especially under 70 °C (Figure 7c).

On the other hand, microstructural change is one of the most significant changes (desirable/undesirable) occurring during the drying process and is closely associated with the quality and storage stability of the final product [45]. SEM imaging helps us know better about surface changing also shrinkage in dried product. As a fact, degrading during food drying, one of the main physical results is shrinkage, which can have unfavorable impacts on the food’s texture, capacity to rehydrate, and surface qualities [46]. Previously, other researchers reported correlation between shrinkage and drying methods, for example, SEM imaging for cherry showed higher shrinkage under hot air drying [47]. The higher shrinkage could be due to a higher moisture gradient that occurred in hot air drying. The higher moisture gradient caused microstructure stress, collapsing of capillary, and irreversible structural change [48]. In another study on dried banana slices, they found that the structure of dried bananas was strongly affected by the drying temperature; as the drying temperature increased, the texture of the crop hardened. This stiffness and fragility of the tissue were observed at high temperatures (80 and 90 °C). This may be due to tissue loosening at high drying temperatures and possibly increased porosity and less tissue contraction [49]. The heat and humidity gradient can cause cell wall destruction, deformation, and shrinkage during the drying process. Deng and Zhao [50] further reported that surface tension and environmental stress may be linked to structural deformation, shrinkage, and collapse of cell structure.

### 3.5. Effective Moisture Diffusivity (D_eff_)

The D_eff_ of the seedless barberry by different drying methods was calculated by Aghbashlo and Samimi-Akhijahani [26] methods. Table 3 demonstrates that the highest D_eff_ was observed in the samples treated by the fastest drying method (Microwave 270 W), which is 9.13 × 10^−9^ m^2^/s; while the lowest value was for the slowest method (i.e., shade), which is 9.68 × 10^−12^ m^2^/s). It was observed with increasing power of microwave D_eff_ also increased. Another researcher proved Moreover, the application of the microwave can elevate D_eff_, compared to the shade, sun, oven and cabinet methods, because the microwave power can increase the molecular movements of the water molecules and enhance the D_eff_ [51].

According to the previous studies, the D_eff_ lies in the range of the 10^−12^–10^−6^ m^2^/s [52]. In agreement with our result, microwave drying yield the highest D_eff_ values which were approximately higher than that of sun and oven drying, for example Arslan et al. [53] dried peppermint using various methods (microwave, oven and sun). According to their results, the D_eff_ of the samples dried by microwave was 4.09 × 10^−10^, while D_eff_ was 3.10 × 10^−12^ and 2.68 × 10^−10^ m^2^/s for those dried by oven and sun respectively. In the work of Altay et al. [52], the D_eff_ of purple basil ranged from 1.42 × 10^−9^ to 5.78 × 10^−8^ m^2^/s for various methods (sun, freeze, hot air and microwave). The D_eff_ of green microalgae dried by various hot airs (sun, solar, hot air and microwave) ranged from 4.64 × 10^−10^ m^2^/s for open sun to 3.91 × 10^−9^ m^2^/s for microwave [54].

### 3.6. Specific Energy Consumption (SEC)

Results showed that SEC was affected by drying methods in seedless barberry. The estimated SEC for the various drying techniques (microwave, oven and cabinet) for the reduction of seedless barberry water is also provided in Table 4. According to result, it can be seen that the SEC for microwave, oven and cabinet was 12.20–246.57 MJ/kg, 358.22–407 MJ/kg and 960.85–1182.14 MJ/kg, respectively. The lowest SEC was related to the microwave. The results of a study by Kaveh et al. [55] on green pea confirmed our result and the microwave had the lowest SEC compared to others. The use of microwave increased thermal gradient and subsequently drying time and as a result, the crops’ moisture removal process was quickened. The decreasing drying time strongly affects SEC [32]. The highest SEC was reported in the cabinet compared to others. Altay et al. [52], Kaveh et al. [55] and Osae et al. [7] obtained similar results.

### 3.7. Energy Efficiency (EE)

The EE for drying seedless barberry varied from 1.16 to 25.26%. Table 4 shows that the 270 W microwave has the highest EE (25.26%) and the lowest value (1.16%) to the cabinet 50 °C. Energy efficiency increases with increasing microwave power and air temperature. Under microwave treatment, the rate of moisture removal from the drying chamber’s interior improved, reducing the drying time for the product and, as a result, the SEC for the drying process, increasing EE [32]. Increasing in drying time of cabinet dryer and oven is the main reason for the high-energy consumption and low EE [33]. These results agree with Kaveh et al. [56] for drying of green pea in various dryers and Torki-Harchegani et al. [57] for peppermint leaves in different dryers.

## 4. Conclusions

The outcomes of this experiment demonstrate that new methods compared to traditional ones speed up the drying and processing of seedless barberry fruit and also increase the amount of vitamin C, TSS, TA, and anthocyanin phenolic compounds in these fruits. It seems that the microwave method, especially in high power (270 W) was effective in drying time, energy efficiency, effective moisture diffusivity coefficient and phenolic compounds. Also, wrinkling and surface damage of the seedless barberry was less than others. Although under sun and shade treatments in storage period, there is a sharper decrease in the color factors, due to the primary preservation of color characteristics compared to others, they were more successful in preserving color characteristics, the reason for this can be attributed to the presence of the heat factor in artificial drying methods, which is high and destruction of seedless barberry pigments plays a role. Therefore, according to the purpose of using seedless barberry fruit (medicinal application, food industry, food seasoning, coloring, and flavoring, etc.) and the priority on the effective substance, suitable methods of drying seedless barberry fruit can be used.

## Figures and Tables

**Table 1:** Treatments include drying methods and storage in this study.

Treatments (Drying Methods)	Storage		
	Before		After
	Microwave (M)	100 W	Kept all dried samples in polyethylene packaging for 6 months under 5–10 °C.
		170 W	
		270 W	
	Cabinet (H)	50 °C	
		70 °C	
	Oven (O)	60 °C	
		70 °C	
	Shade	−	
	Sun	−	

**Table 2:** ANOVA analysis of drying methods and storage on some color indexes.

S.O.V	df	a*	b*	L*	ab	Chroma
Drying method (D)	9	591.35 *	45.65 *	22.61 *	2.95 *	619.65 *
Storage (S)	1	466.09 *	50.87 *	17.94 *	1.07 *	535.15 *
D × S	9	128.48 *	3.49 *	0.48 *	1.35 *	125.48 *
Error	40	0.22	0.15	0.19	0.04	0.21

*: significant at the 5% probability level; Drying methods such as: Microwave 100 W (M 100), Microwave 170 W (M 170), Microwave 270 W (M 270), Oven 60 °C (O 60), Oven 70 °C (O 70), Cabinet 50 °C (H 50), Cabinet 70 °C (H 70) and control (fresh sample). S.O.V; Source of variation.

**Table 3:** ANOVA analysis of drying methods and storage on some biochemical traits.

S.O.V	df	pH	TA	TSS	Vit C	Anthocyanin	Phenol
Drying method (D)	9	0.047 *	1.65 *	12.46 *	127.83 *	24.35 *	1720.19 *
Storage (S)	1	0.003 ns	42.12 *	57.48 *	2317.57 *	50.83 *	18,474.09 *
D × S	9	0.027 *	0.15 *	7.62 ns	111.95 *	27.46 *	781.39 ns
Error	40	0.05	0.16	1.78	2.69	2.62	21.88

*: significant at the 5 % probability level ns: not significant; Drying methods such as: Microwave 100 W (M 100), Microwave 170 W (M 170), Microwave 270 W (M 270), Oven 60 °C (O 60), Oven 70 °C (O 70), Cabinet 50 °C (H 50), Cabinet 70 °C (H 70) and control (fresh sample). S.O.V; Source of variation.

**Table 4:** Energy efficiency, Specific energy consumption (SEC), and Effective moisture diffusivity coefficient (D_eff_) amounts under different drying methods.

Treatment		EE (%)	SEC (MJ/kg)	D_eff_ (m^2^/s)
Microwave	100 W	5.68	246.5753	1.40 × 10^−10^
	170 W	24.68	12.57534	5.59 × 10^−09^
	270 W	25.26	12.20548	9.13 × 10^−09^
Cabinet	50 °C	1.16	1182.141	7.87 × 10^−11^
	70 °C	2.01	960.8532	1.53 × 10^−10^
Oven	60 °C	4.15	407.6347	2.19 × 10^−10^
	70 °C	4.89	358.2235	3.29 × 10^−10^
Shade	−	−	−	9.68 × 10^−12^
Sun	−	−	−	2.73 × 10^−11^

## Data Availability

The data presented in this study are available on request from the corresponding author.
